# Metabolic dysbiosis as a predictor of immunological non-response among people living with HIV

**DOI:** 10.3389/fcimb.2026.1785624

**Published:** 2026-05-15

**Authors:** Heping Zhao, Fenqi Da, Huijun Hou, Xiejie Chen, Xuemei Ling, Shuizhen Wu, Linghua Li

**Affiliations:** 1 Guangzhou Medical Research Institute of Infectious Diseases, Guangzhou Eighth People’s Hospital, Guangzhou Medical University, Guangzhou, China; 2 Infectious Disease Center, Guangzhou Eighth People’s Hospital, Guangzhou Medical University, Guangzhou, China; 3 Guangzhou Center for Disease Control and Prevention, Guangzhou, China

**Keywords:** art, cytokines, immunologic non-response, metabolites, PLWH

## Abstract

**Introduction:**

The molecular mechanisms of immunological non-response in people living with HIV (PLWH) on antiretroviral therapy (ART) remain unclear. This study aimed to identify baseline metabolic and cytokine profiles that differ between immunological responders (IR) and immunological non-responders (INR), thereby exploring early molecular features associated with this discordant outcome.

**Methods:**

A cohort of PLWH was established at Guangzhou Eighth People’s Hospital during November-December 2023. Following a minimum of four years on ART, participants were classified as INR (CD4+ T cell count < 350 cells/μL) or IR (≥350 cells/μL). Baseline plasma metabolites and cytokines were quantified using ultra-performance liquid chromatography-tandem mass spectrometry (UPLC-MS) and the Olink multiplex proximity extension assay (PEA), respectively.

**Results:**

A total of 101 PLWH were enrolled, comprising 46 IR and 55 INR. Baseline CD4+ T cell counts were comparable between the groups (IR: median 116 cells/μL; INR: 82 cells/μL). None of the 92 cytokines showed statistically significant differences between IR and INR. Quantitative analysis of 189 plasma metabolites revealed six with significant differential abundance between the IR and INR groups. Specifically, the INR group exhibited higher concentrations of arginine, glycodeoxycholate acid, glycodeoxycholic acid 3-sulfate, guanidoacetic acid, and tidiacic acid, along with a lower concentration of 2-methylpentanoic acid relative to the IR group. The six differential metabolites were used to construct exploratory classification models. The logistic regression model based on these metabolites yielded an AUC of 0.717 (95% CI: 0.616–0.817). Notably, in both random forest (RF) and support vector machine (SVM) analyses, 2-methylpentanoic acid and guanidoacetic acid were consistently ranked as the top two features for distinguishing INR from IR. Furthermore, correlation analysis indicated that guanidoacetic acid, glycodeoxycholate acid, and glycodeoxycholic acid 3-sulfate levels were inversely associated with the ΔCD4+ T cell count (r = -0.31, -0.38, and -0.35, respectively), whereas 2-methylpentanoic acid showed a positive correlation (r = 0.26).

**Conclusions:**

Divergent immunological responses in PLWH were associated with distinct baseline metabolic profiles. These findings suggest potential metabolic pathways that may contribute to immune non-response and highlight specific plasma metabolites as candidates for future mechanistic and translational studies.

## Introduction

The introduction of antiretroviral therapy (ART) has fundamentally changed the trajectory of HIV infection, transforming what was once a universally fatal illness into a preventable chronic disease (Su et al., 2023; Kelley et al., 2025; Ramgopal et al., 2025). Current data show that most people living with HIV (PLWH) achieve significant virological suppression and robust CD4+ T cell recovery after starting ART. However, a substantial proportion (10-20%) of patients who adhere to treatment fail to regain normal immune function, even when viral replication is controlled, and continue to exhibit low CD4+ T cell counts in the long term (Zhang et al., 2023; Chen et al., 2025). These individuals, termed immunological non-responders (INR), differ markedly from immunological responders (IR), who restore adequate CD4+ T cell levels under effective ART (Guedes et al., 2025). Of clinical importance, INR patients with persistently low CD4+ T cell counts face elevated risks of opportunistic infections, cardiovascular and metabolic diseases, neurocognitive decline, and cancers (Lin et al., 2022; Kelebie et al., 2025; Li et al., 2025; Wei et al., 2025). Crucially, the mechanisms underlying this incomplete immune recovery in INR patients remain largely unclear, posing a major unresolved challenge in HIV management and research.

Consequently, the early identification of INR is imperative to enable timely intervention and improve clinical outcomes. Previous studies have identified that older age at baseline, comorbid opportunistic infections, HBV/HCV co-infection, and a lower CD4+ T cell count are risk factors for INR (Li et al., 2019; Ge et al., 2023). In particular, a lower CD4+ T cell count has been widely confirmed as a significant risk factor for INR. However, we have also observed an interesting phenomenon that some PLWH with a low baseline CD4+ T cell count still achieve good CD4+ T cell recovery after treatment (Zhao et al., 2024). This suggests that, in addition to CD4+ T cell count, other potential and as yet unidentified risk factors may influence immune recovery. Given that CD4+ T cell count is a cellular-level marker, the question arises: could there exist unknown biomarkers at the molecular level?

High-throughput omics analyses, such as metabolomics, proteomics, and transcriptomics, offer excellent tools for identifying molecular markers and have been widely applied in the diagnosis and prediction of diseases. In this context, Bai et al. analyzed microarray datasets and identified six genes (FAM179B, FAM120AOS, JUN, LTA, PTMA, and SH3YL1) as potential key factors implicated in INR (Bai et al., 2021). Given that INR is an immune-related disorder and that metabolism is closely intertwined with the immune system, this study aims to perform integrated targeted metabolomics and proteomics analyses in PLWH with matched baseline CD4+ T cell levels, to explore whether early biomarkers of metabolites or inflammatory factors are associated with INR. This study is expected to provide insights into the mechanisms underlying INR and facilitate its early diagnosis.

## Methods

### Study population and procedures

The cohort comprised PLWH recruited from the Guangzhou Eighth People’s Hospital. Participants were dichotomized into IR and INR. To be eligible, all individuals must have received ART for >4 years and have had a viral load < 50 copies/mL in the past three years. Those who fulfilled these conditions and had a CD4+ T cell count ≥ 350 cells/μL over the preceding year were classified as IR, whereas those with a count < 350 cells/μL were classified as INR.

Eligible participants were PLWH who: 1) were aged ≥18 years; 2) had received ART for ≥4 years; 3) had a baseline CD4+ T cell count < 350 cells/μL; and 4) maintained an HIV viral load < 50 copies/mL over the preceding three years. Exclusion criteria included: 1) incomplete baseline or follow-up records for HIV viral load or CD4+ T cell count; 2) a follow-up duration of < 4 years; 3) pregnancy; 4) active syphilis (antibody titer >1:8); 5) the presence of serious opportunistic infections.

This research was conducted in accordance with the ethical principles outlined in the Declaration of Helsinki. The study protocol was approved by the Institutional Review Board of Guangzhou Eighth People’s Hospital (Approval No. 202343280). Written informed consent was obtained from all participants prior to their enrollment.

### Data collection

Clinical and demographic information was obtained from the National Free Antiretroviral Treatment Program database. Key variables encompassed the following: 1) Sociodemographic characteristics, such as age, gender, body-mass index (BMI), and marital status. 2) HIV treatment details, including infection route, year of HIV diagnosis, year of ART initiation, ART duration (years), baseline and latest ART regimens, baseline and latest CD4+ T cell counts (cells/μL), baseline and latest CD8+ T cell counts (cells/μL), HIV viral load (copies/mL), history of sulfamethoxazole-trimethoprim (SMZ-TMP) use, baseline comorbidities, and baseline symptoms or signs.

3) Coinfections, including syphilis, hepatitis B virus (HBV), and hepatitis C virus (HCV). 4) Biochemical markers, comprising white blood cell count (WBC), hemoglobin (Hb), platelet count (PLT), glucose (Glu), serum creatinine (Scr), total bilirubin (TBIL), total cholesterol (TCHO), triglyceride (TG), alanine aminotransferase (ALT), aspartate aminotransferase (AST), and AST/ALT ratio.

Baseline comorbidities were defined as the presence of one or more of the following conditions: skin lesions, thrush, hairy leukoplakia, recurrent severe bacterial pneumonia, recurrent severe bacterial infections (excluding pneumonia), chronic herpes simplex virus infection, herpes zoster, toxoplasmic encephalitis, cytomegalovirus infection, extrapulmonary cryptococcosis (including meningitis), pneumocystis jirovecii pneumonia (PCP), disseminated mycosis, Kaposi’s sarcoma, cerebral lymphoma or B-cell non-Hodgkin lymphoma, and esophageal candidiasis. Baseline symptoms or signs were defined as the presence of one or more of the following: fever, cough, expectoration, dyspnea, chest pain, night sweats, diarrhea, nausea, headache, visual impairment, and lymphadenopathy. The CD4+ T cell count, CD8+ T cell count, and CD4+/CD8+ ratio were defined as indicators of disease progression.

Peripheral blood was collected from all participants prior to ART initiation. Targeted metabolomic profiling was performed using the Q300 panel (Metabo-Profile, Shanghai, China), a well-established ultra-performance liquid chromatography-tandem mass spectrometry (UPLC-MS) platform (ACQUITY UPLC-Xevo TQ-S, Waters Corp., Milford, MA, USA) designed for the simultaneous quantification of up to 300 metabolites across multiple biochemical classes (including amino acids, organic acids, fatty acids, bile acids, and short-chain fatty acids). Sample preparation was conducted as follows: 20 µL of plasma was mixed with 120 µL of ice-cold methanol containing internal standards, vortexed, and centrifuged. The supernatant was derivatized, diluted, and centrifuged again before UPLC-MS analysis alongside a derivative standard solution for quantification. Pooled quality control (QC) samples were prepared by mixing equal aliquots of all study samples and injected at regular intervals throughout the analytical run to monitor system stability and reproducibility. Raw data were processed using TMBQ software (Metabo-Profile, Shanghai, China) for peak extraction, alignment, and metabolite quantification. Metabolites with >30% missing values were excluded from analysis; for the remaining metabolites, missing values were imputed using half the minimum detected value. Data were then normalized by total peak area and log-transformed prior to statistical analysis to approximate normal distribution.

Plasma cytokine levels were measured using the Olink Proximity Extension Assay (PEA) technology. In this assay, target proteins are captured by pairs of antibodies, each conjugated to a unique DNA oligonucleotide. When both antibodies bind to the same protein, their oligonucleotides are brought into proximity, hybridize, and are extended by a DNA polymerase to form a protein-specific DNA sequence. This DNA template is then amplified and quantified via qPCR. The resulting qPCR data were processed using the Olink NPX Manager software to generate Normalized Protein Expression (NPX) values for subsequent analysis. Samples that did not meet the predefined quality control criteria were excluded from the analysis. For measurements below the lower limit of detection (LOD), values were estimated using the standard curve equation whenever possible; when estimation was not feasible, these values were considered missing and were not imputed.

### Statistical analysis

Descriptive statistics are presented as counts (percentages) or medians (IQR). To compare the IR and INR groups, categorical variables were analyzed using the Pearson Chi-squared test or Fisher’s exact test, while continuous variables were analyzed with the Wilcoxon rank-sum test. Three machine learning models—logistic regression (LR), support vector machine (SVM) with a radial basis function kernel, and a 500-tree random forest (RF)—were constructed based on the differential features. The dataset was randomly partitioned into a training set and a validation set in a 4:1 ratio, with a fixed random seed set to ensure that the entire modeling process can be reproduced. Hyperparameter tuning was performed on the training set using the tune function, employing ten−fold cross−validation to select the combination of parameters yielding the lowest average prediction error. Optimal hyperparameters were then used to train the final models on the full training set. Feature importance was quantified from these final models: for SVM, the absolute values of the model weights were used; for RF, the mean decrease in Gini impurity was calculated. For model evaluation, discriminative performance was assessed by receiver operating characteristic (ROC) curve analysis, and the area under the curve (AUC) with its 95% confidence intervals (CIs) was calculated. Additionally, Spearman correlation analysis was performed between differential metabolites and disease progression indicators, with results visualized in a chord diagram. Statistical significance was set at *P* < 0.05. All statistical computations and visualizations were performed using R software version 4.4.2, with the following packages: caret, circlize, corrplot, dplyr, e1071, ggplot2, ggprism, ggpubr, ggrepel, plotrix, pROC, randomForest, recipes, reshape2, and tidyr.

## Results

### Participant characteristics

As shown in **Table 1**, the study cohort included 101 participants, comprising 46 IR and 55 INR. At baseline, the median CD4+ T cell counts were 116 cells/μL in the IR group and 82 cells/μL in the INR group, with no statistically significant difference between the two groups. After a median ART duration of 6.59 years in the IR group and 6.21 years in the INR group, the current CD4+ T cell count was significantly higher in the IR group (655 cells/μL) than in the INR group (252 cells/μL).

**Table 1:** Demographics and clinical characteristics (N = 101).

Variables	Overall	IR	INR	*P*-value
Total	101 (100%)	46 (45.5%)	55 (54.5%)	
Gender				
Male	65 (64.4%)	26 (56.5%)	39 (70.9%)	0.133
Female	36 (35.6%)	20 (43.5%)	16 (29.1%)	
Age (years)				
Median (IQR)	36 [30-43]	36 [30-42]	36 [30-46]	0.267
18-29	20 (19.8%)	10 (21.7%)	10 (18.2%)	0.758
30-39	42 (41.6%)	20 (43.5%)	22 (40.0%)	
≥40	39 (38.6%)	16 (34.8%)	23 (41.8%)	
BMI				
Median (IQR)	20.51 [18.61-22.72]	20.31 [18.61-23.15]	20.55 [18.51-22.06]	0.948
<18.5	24 (23.8%)	11 (23.9%)	13 (23.6%)	0.624
18.5-24	63 (62.4%)	27 (58.7%)	36 (65.5%)	
≥24	14 (13.9%)	8 (17.4%)	6 (10.9%)	
Marital status				0.268
Married	50 (49.5%)	20 (43.5%)	30 (54.5%)	
Unmarried or divorced	51 (50.5%)	26 (56.5%)	25 (45.5%)	
Infection route				0.615
Homosexual	36 (35.6%)	15 (32.6%)	21 (38.2%)	
Heterosexual	57 (56.4%)	26 (56.5%)	31 (56.4%)	
Others	8 (7.9%)	5 (10.9%)	3 (5.5%)	
Year of HIV diagnosis				0.086
Before 2016	33 (32.7%)	11 (23.9%)	22 (40.0%)	
After 2016	68 (67.3%)	35 (76.1%)	33 (60.0%)	
Time since HIV diagnosis (years)				
Median (IQR)	6.92 [5.25-8.63]	6.95 [5.30-8.25]	6.88 [5.06-8.94]	0.935
<10	87 (86.1%)	41 (89.1%)	46 (83.6%)	0.426
≥10	14 (13.9%)	5 (10.9%)	9 (16.4%)	
Year of ART initiation				0.065
Before 2016	24 (23.8%)	7 (15.2%)	17 (30.9%)	
After 2016	77 (76.2%)	39 (84.8%)	38 (69.1%)	
ART duration (years)				
Median (IQR)	6.48 [5.19-7.78]	6.59 [5.27-7.78]	6.21 [5.00-8.26]	0.989
<5	23 (22.8%)	10 (21.7%)	13 (23.6%)	0.821
≥5	78 (77.2%)	36 (78.3%)	42 (76.4%)	
Time before ART (months)				
Median (IQR)	0.76 [0.39-2.30]	0.79 [0.39-3.91]	0.69 [0.39-2.01]	0.881
<1	66 (65.3%)	29 (63.0%)	37 (67.3%)	0.895
1-6	16 (15.8%)	8 (17.4%)	8 (14.5%)	
≥6	19 (18.8%)	9 (19.6%)	10 (18.2%)	
Baseline ART regimens				0.560
NRTIs+NNRTIs	67 (66.3%)	30 (65.2%)	37 (67.3%)	
NRTIs+PIs	18 (17.8%)	10 (21.7%)	8 (14.5%)	
NRTIs+INSTIs	16 (15.8%)	6 (13.0%)	10 (18.2%)	
Baseline SMZ-TMP				0.285
Yes	8 (7.9%)	2 (4.3%)	6 (10.9%)	
No	93 (92.1%)	44 (95.7%)	49 (89.1%)	
Baseline comorbidities				0.662
Yes	24 (23.8%)	10 (21.7%)	14 (25.5%)	
No	77 (76.2%)	36 (78.3%)	41 (74.5%)	
Baseline symptoms or signs				0.238
Yes	23 (22.8%)	8 (17.4%)	15 (27.3%)	
No	78 (77.2%)	38 (82.6%)	40 (72.7%)	
Baseline HIV load (copies/ml)	59600 [7065-286500]	250000 [36400-444000]	32900 [6620-104000]	0.201
Baseline CD4+ T cell count (cells/μL)				
Median (IQR)	94 [45-180]	116 [56-205]	82 [23-135]	0.075
<200	80 (79.2%)	34 (73.9%)	46 (83.6%)	0.230
200-350	21 (20.8%)	12 (26.1%)	9 (16.4%)	
Baseline CD8+ T cell count (cells/μL)				
Median (IQR)	589 (335-941)	533 (331-742)	622 (393-1, 053)	0.287
<320	23 (22.8%)	10 (21.7%)	13 (23.6%)	0.697
320-1250	69 (68.3%)	33 (71.7%)	36 (65.5%)	
≥1250	9 (8.9%)	3 (6.5%)	6 (10.9%)	
Baseline HBV				0.366
Positive	12 (11.9%)	4 (8.7%)	8 (14.5%)	
Negative	89 (88.1%)	42 (91.3%)	47 (85.5%)	
Baseline HCV				0.624
Positive	4 (4.0%)	1 (2.2%)	3 (5.5%)	
Negative	97 (96.0%)	45 (97.8%)	52 (94.5%)	
Latest ART regimens				0.438
NRTIs+NNRTIs	62 (61.4%)	30 (65.2%)	32 (58.2%)	
NRTIs+PIs	11 (10.9%)	6 (13.0%)	5 (9.1%)	
NRTIs+INSTIs	28 (27.7%)	10 (21.7%)	18 (32.7%)	
Latest CD4+ T cell count (cells/μL)				
Median (IQR)	390 [247-632]	655 [490-787]	252 [164-337]	**<0.001**
<350	44 (43.6%)	2 (4.3%)	42 (76.4%)	**<0.001**
350-500	19 (18.8%)	10 (21.7%)	9 (16.4%)	
≥500	38 (37.6%)	34 (73.9%)	4 (7.3%)	
Latest CD8+ T cell count (cells/μL)				
Median (IQR)	615 (466-853)	592 (451-683)	690 (469-950)	0.114
<320	2 (2.0%)	0 (0.0%)	2 (3.6%)	0.152
320-1250	93 (92.1%)	45 (97.8%)	48 (87.3%)	
≥1250	6 (5.9%)	1 (2.2%)	5 (9.1%)	

Time before ART, time interval between HIV diagnosis and ART initiation. IR, immunological responders; INR, immunological non-responders; BMI, body-mass index; ART, antiretroviral therapy; NRTIs, nucleoside reverse transcriptase inhibitors; NNRTIs, non-nucleoside reverse transcriptase inhibitors; PIs, protease inhibitors; INSTIs, integrase strand transfer inhibitors; SMZ-TMP, sulfamethoxazole and trimethoprim; HBV, hepatitis B virus. HCV, hepatitis C virus. IQR, interquartile range. Bold values indicate P < 0.05.

No significant differences were observed in demographics (age, gender, BMI) or other baseline parameters between the groups, indicating comparable baseline characteristics (**Table 1**). Additionally, baseline and latest biochemical markers were also comparable between the groups. Details are provided in **Table 2**, **Supplementary Table 1**.

**Table 2:** Baseline bioc*hemic*al markers (N = 101).

Variables	Overall	IR	INR	*P*-value
Total	101 (100%)	46 (45.5%)	55 (54.5%)	
Baseline WBC (10^9/L)				
Median (IQR)	4.68 (3.70-6.01)	5.19 (3.72-6.54)	4.61 (3.65-5.76)	0.528
<3.5	22 (21.8%)	9 (19.6%)	13 (23.6%)	0.928
3.5-9.5	75 (74.3%)	35 (76.1%)	40 (72.7%)	
≥9.5	4 (4.0%)	2 (4.3%)	2 (3.6%)	
Baseline Hb (g/L)				
Median (IQR)	130 (107-149)	128 (98-150)	130 (114-148)	0.415
<130	50 (49.5%)	24 (52.2%)	26 (47.3%)	0.624
130-175	51 (50.5%)	22 (47.8%)	29 (52.7%)	
Baseline PLT (10^9/L)				
Median (IQR)	197 (162-259)	203 (174-257)	193 (154-263)	0.435
<125	13 (12.9%)	4 (8.7%)	9 (16.4%)	0.528
125-350	85 (84.2%)	41 (89.1%)	44 (80.0%)	
≥350	3 (3.0%)	1 (2.2%)	2 (3.6%)	
Baseline Glu (mmol/L)				
Median (IQR)	5.14 (4.64-5.68)	5.05 (4.63-5.68)	5.19 (4.73-5.71)	0.623
<3.9	5 (5.0%)	2 (4.3%)	3 (5.5%)	0.999
3.9-6.1	80 (79.2%)	37 (80.4%)	43 (78.2%)	
≥6.1	16 (15.8%)	7 (15.2%)	9 (16.4%)	
Baseline Scr (µmol/L)				
Median (IQR)	69 (59-79)	69 (56-78)	69 (60-79)	0.675
<57	24 (23.8%)	12 (26.1%)	12 (21.8%)	0.616
57-111	77 (76.2%)	34 (73.9%)	43 (78.2%)	
Baseline TBIL (µmol/L)				
Median (IQR)	8.35 (6.27-11.74)	7.98 (5.74-10.77)	8.76 (6.65-11.92)	0.203
<5.1	13 (12.9%)	7 (15.2%)	6 (10.9%)	0.418
5.1-22.2	80 (79.2%)	37 (80.4%)	43 (78.2%)	
≥22.2	8 (7.9%)	2 (4.3%)	6 (10.9%)	
Baseline TCHO (mmol/L)				
Median (IQR)	3.86 (3.21-4.40)	4.03 (3.30-4.67)	3.77 (3.13-4.30)	0.205
<5.18	89 (88.1%)	39 (84.8%)	50 (90.9%)	0.343
≥5.18	12 (11.9%)	7 (15.2%)	5 (9.1%)	
Baseline TG (mmol/L)				
Median (IQR)	1.50 (1.06-2.03)	1.67 (1.08-2.28)	1.41 (0.97-1.93)	0.112
<1.7	62 (61.4%)	24 (52.2%)	38 (69.1%)	0.082
≥1.7	39 (38.6%)	22 (47.8%)	17 (30.9%)	
Baseline ALT (U/L)				
Median (IQR)	25 (18-45)	30 (18-60)	23 (17-36)	0.190
<9	5 (5.0%)	2 (4.3%)	3 (5.5%)	0.820
9-50	73 (72.3%)	32 (69.6%)	41 (74.5%)	
≥50	23 (22.8%)	12 (26.1%)	11 (20.0%)	
Baseline AST (U/L)				
Median (IQR)	25 (20-42)	25 (20-48)	26 (19-36)	0.891
<15	7 (6.9%)	6 (13.0%)	1 (1.8%)	0.061
15-40	68 (67.3%)	27 (58.7%)	41 (74.5%)	
≥40	26 (25.7%)	13 (28.3%)	13 (23.6%)	
Baseline AST/ALT				
Median (IQR)	1.06 (0.77-1.36)	0.99 (0.74-1.30)	1.09 (0.84-1.50)	0.111
<0.8	29 (28.7%)	16 (34.8%)	13 (23.6%)	0.219
0.8-1.5	52 (51.5%)	24 (52.2%)	28 (50.9%)	
≥1.5	20 (19.8%)	6 (13.0%)	14 (25.5%)	

IR, immunological responders; INR, immunological non-responders; WBC, white blood cell; Hb, hemoglobin; PLT, platelet; Glu, glucose; Scr, serum creatinine; TBIL, total bilirubin; TCHO, total cholesterol; TG, triglyceride; ALT, alanine aminotransferase; AST, aspartate aminotransferase; IQR, interquartile range.

### Identification of metabolite signatures

**Figure 1:**
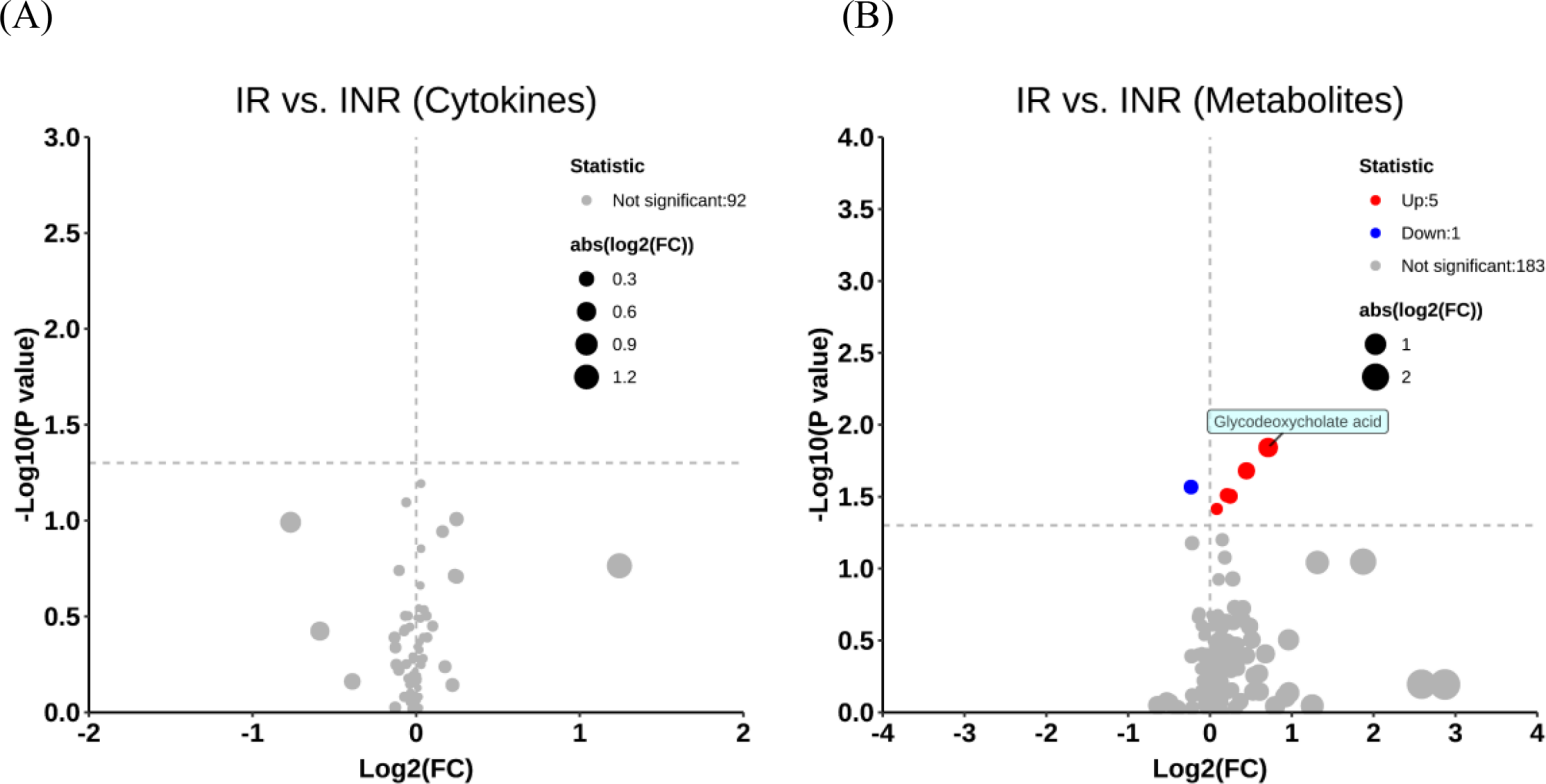
Concentration profiles of cytokines and metabolites (N = 101).

**Figure 2:**
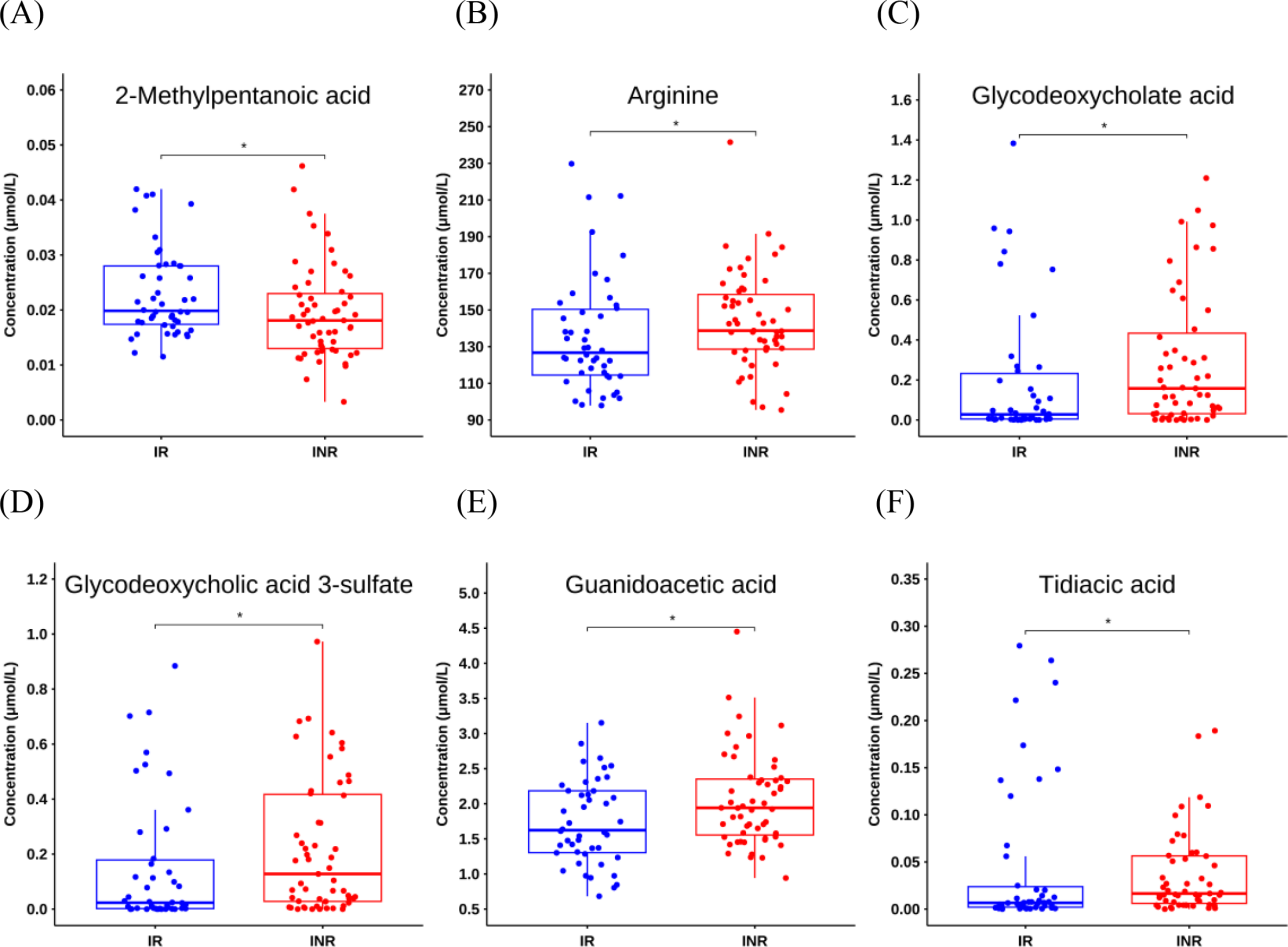
The levels of differential metabolites (N = 101). Statistical differences were evaluated using Wilcoxon rank-sum test. *0.01 ≤ P < 0.05.

A total of 92 cytokines were quantified, but no significant differences were observed between the IR and INR groups (**Figure 1A**). We then profiled the metabolome. Among 189 annotated metabolites, six showed statistically significant alterations in the INR group compared to the IR group (**Figure 1B**). Specifically, the level of 2-methylpentanoic acid was downregulated, while the levels of arginine, glycodeoxycholate acid, glycodeoxycholic acid 3-sulfate, guanidoacetic acid, and tidiacic acid were upregulated in the INR group compared to the IR group (**Figure 2**; **Supplementary Table 2**).

**Figure 3:**
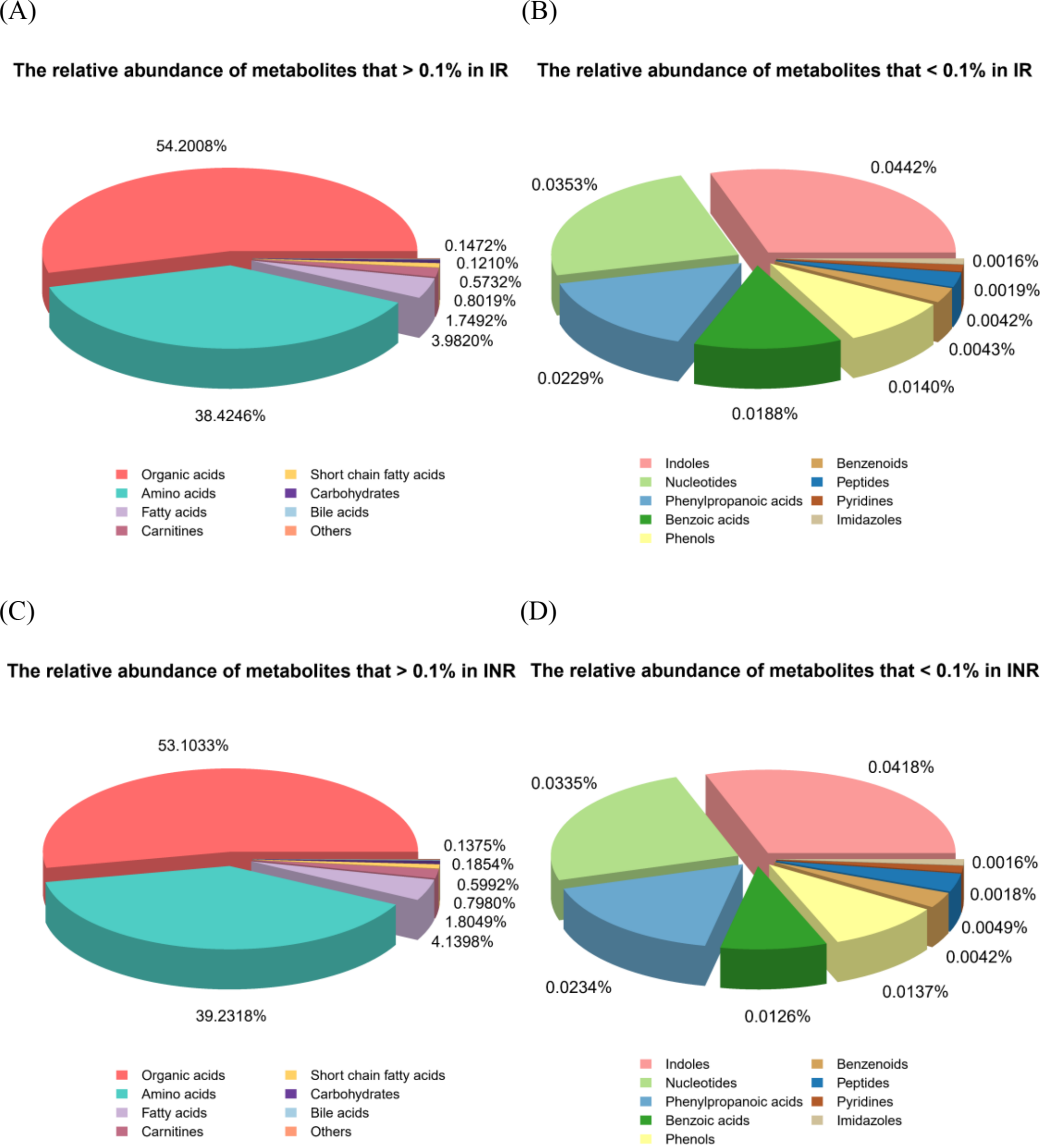
The ranking of metabolite classes (N = 101).

We then investigate whether the hierarchical ranking of metabolites was aligned among the different groups. All identified plasma metabolites were classified into 16 categories (**Figure 3**). The ranking of high-abundance metabolites (>0.1% relative abundance) was largely consistent across groups, including organic acids, amino acids, fatty acids, carnitines, short chain fatty acids, carbohydrates, and bile acids (**Figures 3A, C**). However, the order of low-abundance metabolites (<0.1%) showed minor variations. For example, benzoic acids (0.0188% of total metabolites) ranked as the sixth lowest in the IR group but fell to the fifth lowest in the INR group (0.0126%). Conversely, peptides (0.0042%) ranked as the third lowest in the IR group but rose to the fourth lowest in the INR group (0.0049%) (**Figures 3B, D**).

### Discriminative analysis of immunological response status

**Figure 4:**
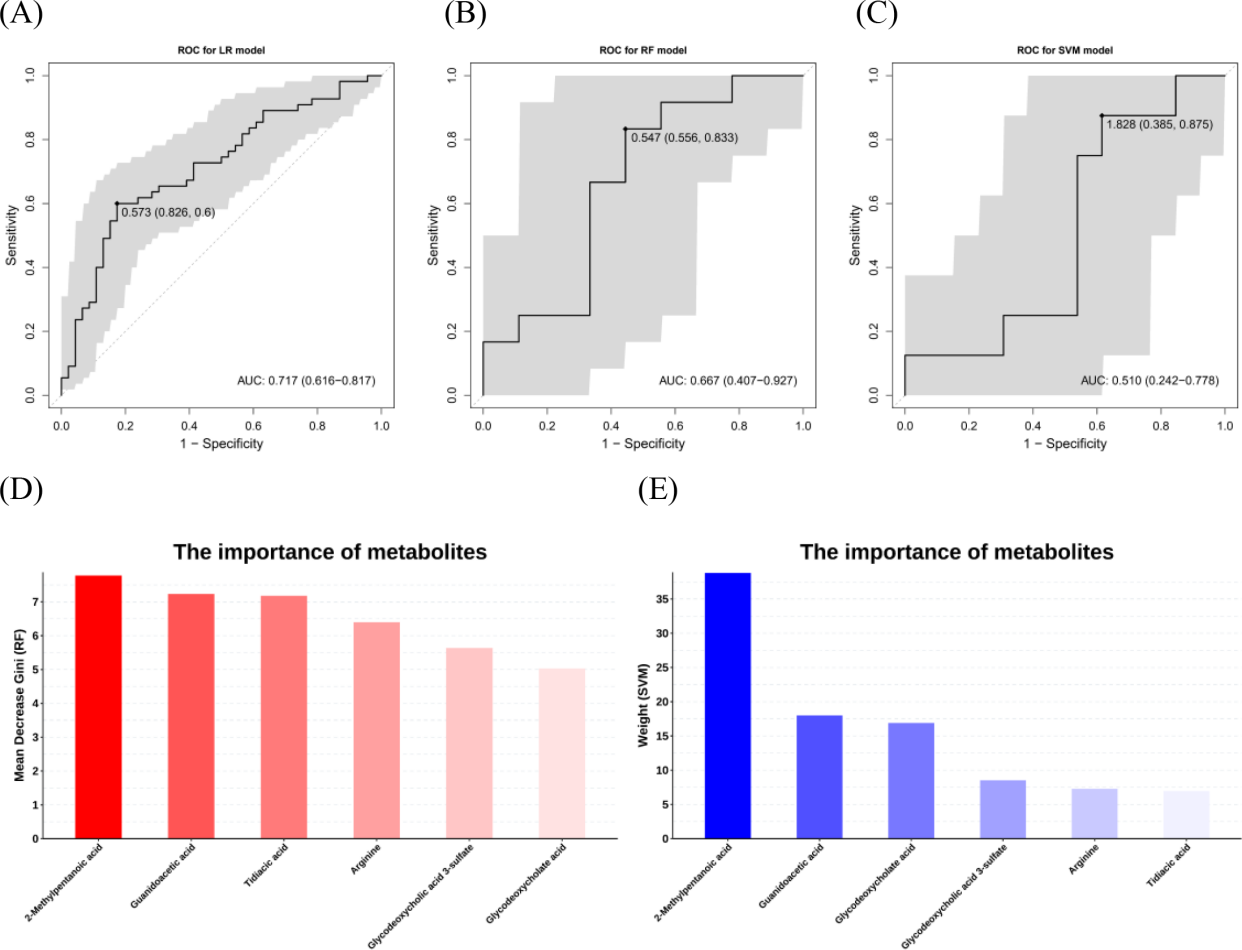
The diagnostic evaluation of the metabolite signatures. **(A–C)** The ROC curve based on six differential metabolites for LR model, RF model and SVM model, respectively. Diagonal lines represent random classification (AUC = 0.5). **(D, E)** The importance of metabolites in discrimination between IR and INR based on RF and SVM analyses, respectively. ROC, receiver operating characteristic curve; AUC, area under the curve; LR, Logistic regression, RF, random forest; SVM, support vector machine.

To rigorously evaluate the discriminatory power of the differential metabolite panel, we employed a multiparametric machine−learning approach incorporating three distinct algorithms: LR, RF, and SVM. The diagnostic performance of each model was assessed using ROC curve analysis, with the AUC serving as the primary metric. Comparative analysis of the models indicated that the LR classifier exhibited the strongest discriminative capacity, achieving the highest AUC of 0.717 (95% CI: 0.616–0.817) (**Figures 4A–C**). In addition to overall model performance, we also examined metabolite importance rankings generated by the RF and SVM algorithms. Notably, 2-methylpentanoic acid and guanidoacetic acid were consistently identified as the two most influential features in distinguishing between the IR and INR groups (**Figures 4D, E**). These findings not only validate the differential metabolite panel but also highlight specific metabolites that may play key roles in the metabolic divergence underlying immunological non−response.

### Correlations

**Figure 5:**
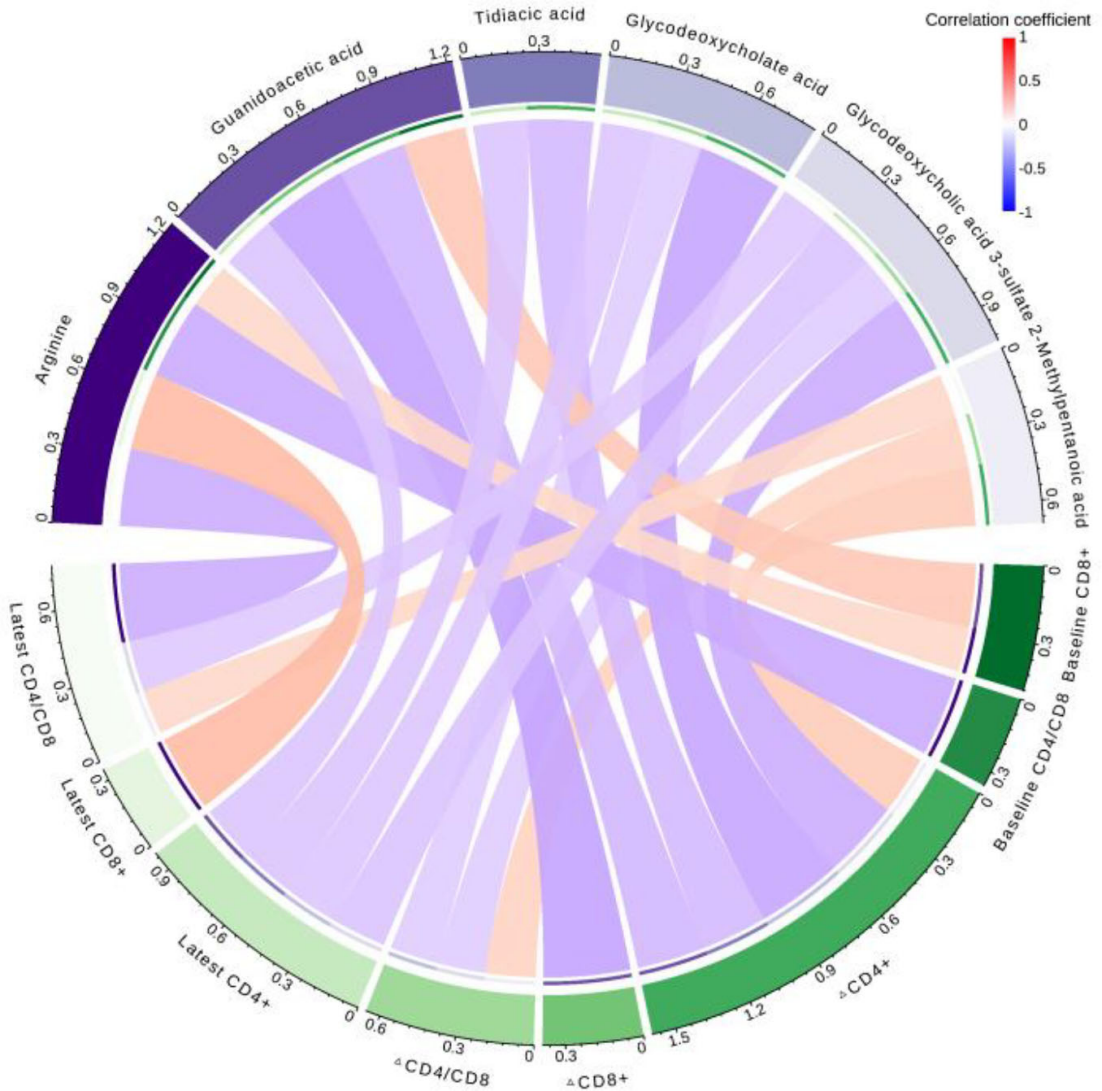
Correlations (N = 101). Correlations between differential metabolites and indicators of disease progression.

We further performed Spearman correlation analysis to examine whether changes in the above six differential metabolites were associated with indicators of disease progression. Our analysis showed that the levels of guanidoacetic acid, glycodeoxycholate acid, and glycodeoxycholic acid 3-sulfate were inversely correlated with the ΔCD4+ T cell count (r = -0.31, -0.38, and -0.35, respectively) (**Figure 5**). Additionally, guanidoacetic acid was negatively correlated with the ΔCD8+ T cell count (r = -0.39) (**Figure 5**). Notably, we found that 2-methylpentanoic acid exhibited a positive correlation with both ΔCD4+ T cell count (r = 0.26) and ΔCD4+/CD8+ ratio (r = 0.22) (**Figure 5**).

## Discussion

This study presents a systematic metabolomic and proteomic profiling of PLWH who exhibited divergent immunological outcomes following long-term ART. It identifies a panel of specific plasma metabolites closely linked to immune reconstitution status. Within a well-characterized cohort with comparable baseline demographics and clinical parameters, we discovered six metabolites that were significantly altered in INR compared to IR. Among these, 2-methylpentanoic acid was downregulated, while arginine, glycodeoxycholate acid, glycodeoxycholic acid 3-sulfate, guanidoacetic acid, and tidiacic acid were upregulated. A multi-parameter machine-learning approach confirmed the ability of this metabolite panel to distinguish between IR and INR, with 2-methylpentanoic acid and guanidoacetic acid emerging as the most influential features. Importantly, correlation analyses revealed significant associations between the levels of these metabolites and indicators of disease progression. Specifically, guanidoacetic acid inversely correlated with ΔCD4+ T cell count, whereas 2-methylpentanoic acid showed positive correlations with both ΔCD4+ T cell count and the ΔCD4+/CD8+ ratio. These findings suggest that distinct metabolic reprogramming is linked to impaired immune reconstitution despite prolonged ART, highlighting potential metabolic contributors to the pathophysiology of immunological non-response.

Previous research on factors influencing INR has primarily focused on macroscopic clinical indicators, such as baseline CD4+ T cell count, age, and co-infections (Li et al., 2019; Zhao et al., 2024), with limited investigation into molecular-level changes. In this study, we performed multi-omics profiling in well-matched groups of IR and INR individuals with comparable baseline CD4+ T cell counts and other clinical parameters. Interestingly, our results showed no statistically significant differences in baseline inflammatory cytokine levels between the two groups. Excessive inflammatory responses have long been implicated in driving heightened pyroptosis of CD4+ T cells in INR (Xia et al., 2023; Kießling et al., 2024; Vos et al., 2024). A recent study from our team also reported severe dysregulation of inflammatory markers, including PD-L1 and FGF-19, in PLWH who experienced suboptimal immune recovery after several years of ART (Zhao et al., 2025). It is therefore puzzling how to account for the absence of differences in baseline inflammatory markers observed in the present study. We hypothesize that the absence of significant differences in baseline inflammatory factors may be due to the comparable baseline levels of CD4+ and CD8+ T cells between the groups, since inflammatory cytokines are typically secreted by immune cells (Bhol et al., 2024). This further suggests that inflammatory factors and key disease progression indicators, such as CD4+ T cell count, may change synchronously during the development of immune non-response, rather than preceding the decline in CD4+ T cell count. Naturally, this hypothesis requires validation through longitudinal sampling and analysis in future studies. It is also important to acknowledge that the statistical power to detect subtle differences may have been limited by our cohort size. Furthermore, while all samples were collected prior to ART initiation (and thus were not subject to its potential suppressive effect on systemic inflammation), the single time-point design cannot account for potential dynamic fluctuations.

Interestingly, although no significant differences were observed in inflammatory cytokine levels between the groups, distinct variations were evident in baseline metabolite profiles. Notably, we found altered rankings in the relative abundance of classified metabolites among the INR group. Specifically, compared to the IR group, the relative abundance ranking of benzoic acids decreased, while that of peptides increased in the INR group. This suggests that, beyond the six differential metabolites identified in this study, many other metabolites may be near the threshold of statistical significance. Of particular interest is the phenomenon of metabolic disturbance preceding inflammatory dysregulation, which offers a new perspective on the role of the metabolic-immune axis in HIV infection progression. Although metabolic reprogramming of immune cells in HIV has been extensively reported (Muñoz-Muela et al., 2024; Ayrga and Koorsen, 2025; Chan et al., 2025; Perdomo-Celis et al., 2025), our findings indicate that metabolic disturbances established early in infection may subtly influence immune reconstitution trajectories before a marked increase in systemic inflammatory markers becomes apparent. Such metabolic differences may constitute a predisposing factor or early risk basis for INR. Therefore, interventions targeting the metabolic-immune axis could represent a promising therapeutic window earlier than current inflammatory targets. Future studies should further investigate how these specific differential metabolites contribute to the mechanisms of immune non−response by affecting CD4+ T cell metabolism, function, or survival. Furthermore, it should be noted that metabolic disturbance represents a persistent pathological state throughout the course of HIV infection and is difficult to fully correct with ART alone (Deme et al., 2022; Feng et al., 2025). Given that the characteristics of metabolic disorders vary across different disease stages, addressing metabolic dysregulation as an independent therapeutic target, while working toward immune reconstitution, has become a critical component of clinical management.

Using multiple machine learning algorithms, we identified 2-methylpentanoic acid as the most important metabolite among the six differential metabolites for distinguishing different immune statuses. 2-methylpentanoic acid belongs to the class of short chain fatty acids (SCFAs), which are produced by the bacterial anaerobic fermentation of carbohydrates (Parada Venegas et al., 2019). We note that our study demonstrates an association, not a mechanism. However, this finding invites mechanistic hypotheses based on established biology. SCFAs are known to modulate host defense by regulating innate immune cell responses and can influence adaptive immunity, such as by regulating the differentiation and function of regulatory T cells (Tregs) (Enriquez et al., 2023). Yu et al. previously reported a reduced abundance of SCFA-producing bacteria in INR and demonstrated *in vitro* that SCFAs supplementation could restore Treg function, enhancing immunosuppressive capacity and improving cellular metabolism (Yu et al., 2025). This suggests that, alongside direct dietary SCFAs intervention (Brauckmann et al., 2022), modulating gut microbial homeostasis could potentially serve as a strategy to regulate host metabolic balance and ultimately assist in immune reconstitution for INR. Our current associative finding of altered 2-methylpentanoic acid levels contributes to this line of inquiry by highlighting a specific metabolic candidate. Consistently, our previous work observed reduced gut microbial diversity and altered abundances of specific genera such as *Coprococcus*, *Blautia*, and *Megamonas* in INR (Zhao et al., 2023). Taken together, these associative observations lead us to hypothesize that the gut microbiota–SCFA–immune axis might be perturbed in INR, with 2-methylpentanoic acid as a potential indicator or mediator. This hypothesis, generated from clinical associations, requires direct experimental validation in future studies to establish any causal link between this metabolite, gut microbes, and immune dysfunction.

In interpreting these findings, the definition of INR itself warrants consideration. The choice of the 350 cells/µL threshold for defining INR in this study was based on established clinical guidelines and its widespread use in the field (Guedes et al., 2025). In our cohort, the median CD4+ T cell count in the INR group was 252 cells/µL (IQR: 164–337), indicating clear separation from the cutoff. We acknowledge that alternative definitions of immunological non-response—such as a stricter cutoff of <200 cells/µL, CD4/CD8 ratio recovery, or ΔCD4 slope—may capture different aspects of immune dysfunction and could provide complementary biological insights. Future studies incorporating multiple definitions may further refine our understanding of metabolic determinants underlying suboptimal immune recovery.

This study has several limitations. Firstly, we did not evaluate the predictive value of differential metabolites in individuals with inconsistent baseline CD4+ T cell counts, nor did we analyze the metabolic profiles of healthy controls (HC), which may limit the generalizability of our findings. Secondly, while machine learning models showed discriminative potential, the moderate AUC (0.717) and the lack of validation in an independent external cohort necessitate cautious interpretation of the predictive utility of the six-metabolite panel. Thirdly, although the targeted metabolomics and cytokine panels were informative, they are not exhaustive; Furthermore, multiple testing correction (e.g., false discovery rate) was not applied to the 189 metabolite comparisons; the findings presented are therefore based on unadjusted *P*-values and should be considered exploratory. Independent validation in larger cohorts is warranted to confirm these preliminary observations. Fourthly, although the groups were well-matched for key clinical parameters, potential residual confounding from unmeasured factors cannot be ruled out. Specifically, we did not assess the detailed dietary intake of participants, which is a recognized factor that can influence both gut microbiota composition and host metabolism, including the levels of short-chain fatty acids and bile acids. Finally, while the study identifies associations, it does not elucidate the underlying biological mechanisms linking specific metabolites, such as 2-methylpentanoic acid or guanidoacetic acid, to impaired CD4+ T cell recovery. Functional validation experiments are needed to confirm their pathogenic roles.

This study compared baseline metabolic and cytokine profiles in PLWH with different immune recovery despite long-term ART. While no cytokine differences were found, six plasma metabolites were significantly altered in INR. Machine learning confirmed these metabolites’ discriminatory power (AUC 0.717), with 2-methylpentanoic acid and guanidoacetic acid as top features. Their levels correlated with ΔCD4+ T cell recovery, suggesting baseline metabolic reprogramming may precede and contribute to immunological non-response. These findings highlight specific plasma metabolites as features associated with immune outcomes and reveal the metabolic-immune axis as a crucial determinant of immune reconstitution, offering new avenues for targeted therapeutic strategies. Collectively, these findings should be viewed as hypothesis-generating rather than clinically definitive, underscoring the need for independent validation and mechanistic studies before any translational application can be considered.

## Data Availability

The raw data supporting the conclusions of this article will be made available by the authors, without undue reservation.
