# Supplementary material for: Metabolic dysbiosis as a predictor of immunological non-response among people living with HIV
Source: Front Cell Infect Microbiol. 2026 May 15;16:1785624. doi: 10.3389/fcimb.2026.1785624 (PMC13219330; doi:10.3389/fcimb.2026.1785624)
Supplement: Supplementary file 1 [file Table1.docx]

Table S1. Latest biochemical markers.

| **Variables** | **Overall** | **IR** | **INR** | ***P*-value** |
| --- | --- | --- | --- | --- |
| **Total** | 101(100%) | 46(45.5%) | 55(54.5%) |  |
| **Latest WBC (10^9/L)** |  |  |  |  |
| Median (IQR) | 5.84 (5.04-7.13) | 6.10 (5.60-7.30) | 5.64 (4.85-6.97) | 0.075 |
| <3.5 | 3 (3.0%) | 0 (0.0%) | 3 (5.5%) | 0.379 |
| 3.5-9.5 | 93 (92.1%) | 44 (95.7%) | 49 (89.1%) |  |
| ≥9.5 | 5 (5.0%) | 2 (4.3%) | 3 (5.5%) |  |
| **Latest Hb (g/L)** |  |  |  |  |
| Median (IQR) | 144 (125-156) | 142 (120-157) | 144 (125-155) | 0.767 |
| <130 | 32 (31.7%) | 15 (32.6%) | 17 (30.9%) | 0.999 |
| 130-175 | 67 (66.3%) | 30 (65.2%) | 37 (67.3%) |  |
| ≥175 | 2 (2.0%) | 1 (2.2%) | 1 (1.8%) |  |
| **Latest PLT (10^9/L)** |  |  |  |  |
| Median (IQR) | 247 (211-287) | 252 (218-310) | 232 (198-281) | 0.112 |
| <125 | 2 (2.0%) | 0 (0.0%) | 2 (3.6%) | 0.253 |
| 125-350 | 86 (85.1%) | 38 (82.6%) | 48 (87.3%) |  |
| ≥350 | 13 (12.9%) | 8 (17.4%) | 5 (9.1%) |  |
| **Latest Glu (mmol/L)** |  |  |  |  |
| Median (IQR) | 5.07 (4.78-5.64) | 5.04 (4.68-5.76) | 5.12 (4.89-5.56) | 0.429 |
| <3.9 | 1 (1.0%) | 1 (2.2%) | 0 (0.0%) | 0.773 |
| 3.9-6.1 | 85 (84.2%) | 38 (82.6%) | 47 (85.5%) |  |
| ≥6.1 | 15 (14.9%) | 7 (15.2%) | 8 (14.5%) |  |
| **Latest Scr (µmol/L)** |  |  |  |  |
| Median (IQR) | 75 (61-91) | 69 (59-90) | 78 (63-93) | 0.121 |
| <57 | 14 (13.9%) | 10 (21.7%) | 4 (7.3%) | **0.049** |
| 57-111 | 81 (80.2%) | 32 (69.6%) | 49 (89.1%) |  |
| ≥111 | 6 (5.9%) | 4 (8.7%) | 2 (3.6%) |  |
| **Latest TBIL (µmol/L)** |  |  |  |  |
| Median (IQR) | 9.70 (7.03-13.10) | 9.16 (7.19-12.61) | 10.34 (6.50-14.21) | 0.551 |
| <5.1 | 11 (10.9%) | 1 (2.2%) | 10 (18.2%) | **0.003** |
| 5.1-22.2 | 86 (85.1%) | 45 (97.8%) | 41 (74.5%) |  |
| ≥22.2 | 4 (4.0%) | 0 (0.0%) | 4 (7.3%) |  |
| **Latest TCHO (mmol/L)** |  |  |  |  |
| Median (IQR) | 4.34 (3.82-5.26) | 4.57 (3.90-5.42) | 4.21 (3.74-5.14) | 0.177 |
| <5.18 | 73 (72.3%) | 30 (65.2%) | 43 (78.2%) | 0.147 |
| ≥5.18 | 28 (27.7%) | 16 (34.8%) | 12 (21.8%) |  |
| **Latest TG (mmol/L)** |  |  |  |  |
| Median (IQR) | 1.26 (0.89-1.98) | 1.27 (0.85-1.98) | 1.25 (0.90-2.20) | 0.653 |
| <1.7 | 69 (68.3%) | 30 (65.2%) | 39 (70.9%) | 0.540 |
| ≥1.7 | 32 (31.7%) | 16 (34.8%) | 16 (29.1%) |  |
| **Latest ALT (U/L)** |  |  |  |  |
| Median (IQR) | 21 (16-29) | 21 (18-33) | 22 (15-27) | 0.411 |
| <9 | 3 (3.0%) | 0 (0.0%) | 3 (5.5%) | 0.364 |
| 9-50 | 87 (86.1%) | 41 (89.1%) | 46 (83.6%) |  |
| ≥50 | 11 (10.9%) | 5 (10.9%) | 6 (10.9%) |  |
| **Latest AST (U/L)** |  |  |  |  |
| Median (IQR) | 23 (19-28) | 23 (18-27) | 22 (19-29) | 0.478 |
| <15 | 4 (4.0%) | 1 (2.2%) | 3 (5.5%) | 0.814 |
| 15-40 | 88 (87.1%) | 41 (89.1%) | 47 (85.5%) |  |
| ≥40 | 9 (8.9%) | 4 (8.7%) | 5 (9.1%) |  |
| **Latest AST/ALT** |  |  |  |  |
| Median (IQR) | 1.01 (0.83-1.40) | 0.96 (0.83-1.22) | 1.11 (0.82-1.49) | 0.134 |
| <0.8 | 23 (22.8%) | 11 (23.9%) | 12 (21.8%) | 0.396 |
| 0.8-1.5 | 59 (58.4%) | 29 (63.0%) | 30 (54.5%) |  |
| ≥1.5 | 19 (18.8%) | 6 (13.0%) | 13 (23.6%) |  |

IR, immunological responders; INR, immunological non-responders; WBC, white blood cell; Hb, hemoglobin; PLT, platelet; Glu, glucose; Scr, serum creatinine; TBIL, total bilirubin; TCHO, total cholesterol; TG, triglyceride; ALT, alanine aminotransferase; AST, aspartate aminotransferase; IQR, interquartile range.

Table S2. The levels of differential metabolites.

| **Variables** | **Overall** | **IR** | **INR** | ***P*-value** |
| --- | --- | --- | --- | --- |
| **Total** | 101(100%) | 46(45.5%) | 55(54.5%) |  |
| **Arginine** |  |  |  |  |
| Median (IQR) | 135.546[120.058-155.130] | 126.738[114.081-151.364] | 138.757[128.137-160.259] | **0.038** |
| **Guanidoacetic acid** |  |  |  |  |
| Median (IQR) | 1.896[1.453-2.326] | 1.624[1.298-2.205] | 1.942[1.531-2.369] | **0.031** |
| **2-Methylpentanoic acid** |  |  |  |  |
| Median (IQR) | 0.019[0.016-0.026] | 0.020[0.017-0.028] | 0.018[0.013-0.023] | **0.027** |
| **Taurodeoxycholic acid** |  |  |  |  |
| Median (IQR) | 0.013[0.004-0.056] | 0.007[0.002-0.033] | 0.017[0.005-0.057] | **0.031** |
| **Glycodeoxycholate acid** |  |  |  |  |
| Median (IQR) | 0.084[0.007-0.325] | 0.028[0.005-0.250] | 0.158[0.030-0.454] | **0.014** |
| **Glycodeoxycholic acid 3-sulfate** |  |  |  |  |
| Median (IQR) | 0.069[0.006-0.314] | 0.023[0.002-0.208] | 0.128[0.028-0.420] | **0.021** |

IR, immunological responders; INR, immunological non-responders; IQR, interquartile range. Unit: μmol/L.
